# Supplementary material for: Towards biologically plausible model-based reinforcement learning in recurrent spiking networks by dreaming new experiences
Source: Sci Rep. 2024 Jun 25;14:14656. doi: 10.1038/s41598-024-65631-y (PMC11199658; doi:10.1038/s41598-024-65631-y)
Supplement: Supplementary file 1 — Supplementary Figure S1. [file 41598_2024_65631_MOESM1_ESM.pdf]

Towards biologically plausible model-based reinforcement learning in recurrent spiking networks by dreaming new experiences

Cristiano Capone[1,2] and Pier Stanislao Paolucci[1]

[1]INFN, Sezione di Roma, Italy RM 00185
[2]Current affiliation: Natl. Center for Radiation Protection and Computational Physics, Istituto Superiore di Sanità, 00161 Rome, Italy

Supplementary Materials

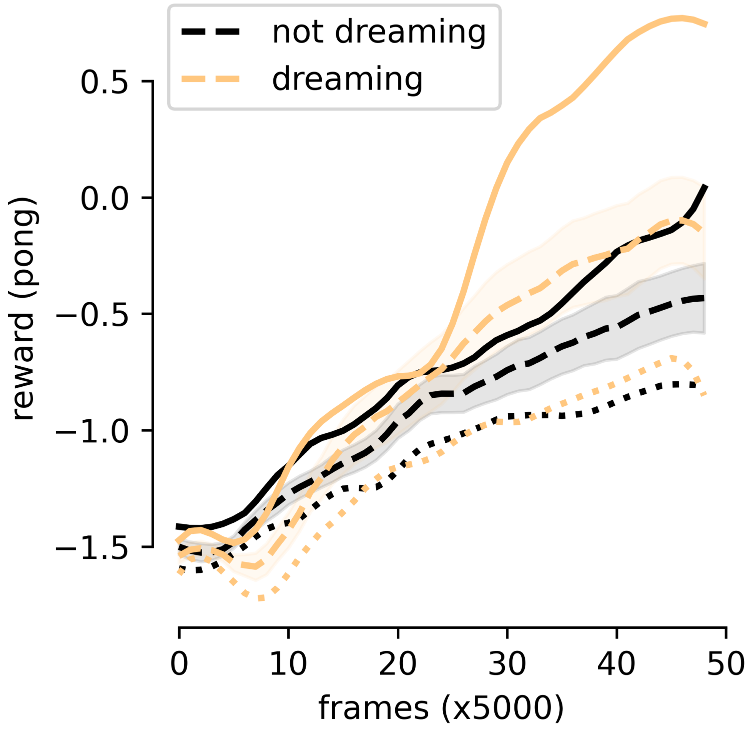

Figure S1: **Learning curve for the atari pong from pixel task.** Average (dashed line), standard error (shading), 80th percentile (solid line), and 20th percentile (dotted line) over 10 independent realizations of the achieved reward, for the atari pong task from pixels. Reward as a function of the number of interactions with the environment, with (orange) and without (black) the dreaming phase.
